# A computational model for the transit of a cancer cell through a constricted microchannel

**DOI:** 10.1007/s10237-023-01705-6

**Published:** 2023-02-28

**Authors:** Z. Wang, R. Lu, W. Wang, F. B. Tian, J. J. Feng, Y. Sui

**Affiliations:** 1 School of Engineering and Materials Science, Queen Mary University of London, London, E1 4NS UK; 2 School of Engineering and Information Technology, University of New South Wales, Canberra, ACT 2600 Australia; 3 Departments of Mathematics and Chemical and Biological Engineering, University of British Columbia, Vancouver, BC V6T 1Z2 Canada

**Keywords:** Biological fluid dynamics, Cancer cells, Microfluidics, Immersed boundary method

## Abstract

We propose a three-dimensional computational model to simulate the transient deformation of suspended cancer cells flowing through a constricted microchannel. We model the cell as a liquid droplet enclosed by a viscoelastic membrane, and its nucleus as a smaller stiffer capsule. The cell deformation and its interaction with the suspending fluid are solved through a well-tested immersed boundary lattice Boltzmann method. To identify a minimal mechanical model that can quantitatively predict the transient cell deformation in a constricted channel, we conduct extensive parametric studies of the effects of the rheology of the cell membrane, cytoplasm and nucleus and compare the results with a recent experiment conducted on human leukaemia cells. We find that excellent agreement with the experiment can be achieved by employing a viscoelastic cell membrane model with the membrane viscosity depending on its mode of deformation (shear versus elongation). The cell nucleus limits the overall deformation of the whole cell, and its effect increases with the nucleus size. The present computational model may be used to guide the design of microfluidic devices to sort cancer cells, or to inversely infer cell mechanical properties from their flow-induced deformation.

## Introduction

The dynamics of cancer cells flowing in microchannels is a fundamental problem that has attracted increasing attention in recent years. The problem is highly relevant to cancer metastasis, where cancer cells shed into the bloodstream from the primary tumour, travel through the blood or lymphatic circulations to distant organs and form secondary tumours. The fluid dynamics of cancer cells is also at the heart of the recent developments of microfluidic technologies for cancer diagnosis and monitoring. It has been well established that the mechanical properties of cancerous cells can be very different from their healthy counterparts (Suresh 2007; Lee and Lim 2007). The difference leads to distinct cell flow trajectories in microchannels, which has been utilized to isolate cancer cells for clinical purposes (Chen et al 2012; Shields IV et al 2015). In the past two decades, considerable efforts have been focused on the mechanical characterization of cancerous and normal cells (Darling and Di Carlo 2015; Wu et al 2018; Guck 2019). Different methods have been proposed, which typically apply well-defined stress to cells and measure the deformation to extract their mechanical properties. Among these methods, the deformability cytometry (DC) (Gossett et al 2012; Byun et al 2013; Otto et al 2015; Mietke et al 2015; Ahmmed et al 2018; Fregin et al 2019; Armistead et al 2019; Urbanska et al 2020) is a promising technique due to its much higher throughput rate than classical methods such as micropipette aspiration and atomic force microscopy. In DC, cells flow through a microfluidic channel and deform under the fluid viscous stress. The deformed cell profiles can then be used to infer cell mechanical properties, which serve as a label-free biomarker to quantify cell states and distinguish cancerous cells from normal ones (Di Carlo 2012; Guck 2019).

Numerical simulations provide an important alternative approach to study the dynamics of cancer cells in flows and they can complement experiments for the following reasons. Firstly, in microfluidics for mechanical characterization of cancer cells (e.g. the DC), experimental measurements must be fitted to model predictions to infer cell mechanical properties. Analytical solutions are always limited to small cell deformation in the Stokes flow regime. Large cell deformation in inertial flows, which is a common feature in high-throughput microfluidics, can only be solved by means of numerical simulations. Secondly, numerical simulations can provide crucial information about cell dynamics that cannot be measured conveniently in experiments, such as the flow fields around and inside individual cells. Thirdly, compared with trial-and-error experiments, numerical simulations enable faster, cheaper, and rational design and optimisation of microfluidic devices for cell sorting or mechanical characterization.

Most previous numerical studies of cell dynamics under flows have been focused on red blood cells (RBCs), which consist of a thin membrane enclosing Newtonian haemoglobin solution (Zhang et al 2008; Krüger et al 2013; Peng et al 2013; Freund 2014; Fedosov et al 2014; Secomb 2017; Balogh and Bagchi 2017; Shen et al 2018). Cancer cells usually have a more complicated structure with a cell membrane, cytoplasm (including the cytosol, cytoskeleton and various organelles) and a nucleus (Lim et al 2006). So far, there have been mainly three types of continuum mechanical models for the dynamics of cancer cells in flows. The first considers cancer cells as single or compound liquid droplets (Leong et al 2011; Zhang et al 2017). Although lacking a membrane, the model can successfully recover the flow velocity of a breast cancer cell when it is entering a constricted microchannel (Leong et al 2011). The second type of models considers a cancer cell as a deformable microcapsule, which consists of a liquid droplet enclosed by a thin elastic membrane (Takeishi et al 2015; King et al 2015; Xiao et al 2016; Cui et al 2021). Although these models have been used to study blood flow with a large number of suspended blood and cancer cells, direct comparison with experiments is rare. The third type of model treats a cancer cell as a compound microcapsule, using a smaller capsule to mimic the cell nucleus (Balogh et al 2021). The cell nucleus has been shown to be crucial in reproducing the shape and passage time of murine lung cancer cells through a microchannel that is smaller than the cells. More details of the cancer cell models can be found from Lim et al (2006); Puleri et al (2021). Notably, active cell migration in extracellular matrix (ECM), on substrates or in confinement has been modelled extensively (Borau et al 2011; Tozluoğlu et al 2013; Allena et al 2015; Zhu and Mogilner 2016; Lee et al 2017; Maxian et al 2020). Our focus here is on cells carried passively by the flow.

Biological cells present viscoelastic behaviour in response to external stresses (Desprat et al 2005; Suresh 2007). The elastic response is mainly due to the cytoskeleton, while the viscous response comes from the membrane lipid bilayer and the cytoplasm. Cancer cells flowing in constricted or cross-slot microchannels in DC may experience strain rates of $10^3$ $\text {s}^{-1}$ or higher. Therefore, it is expected that the viscosity of the subcellular components, particularly the membrane, will play a significant role in determining the dynamics of the cells. For RBCs in micropipette aspiration, the viscous dissipation in the cell membrane can be two orders of magnitude greater than that in the haemoglobin solution (Evans and Hochmuth 1976; Hochmuth and Waugh 1987; Puig-de-Morales-Marinkovic et al 2007). However, most previous numerical studies on the dynamics of cancer cells in microchannels have not taken into account cell membrane viscosity. The only two exceptions we have found are Lykov et al (2017) for breast epithelial cells and Barber and Zhu (2019) for breast cancer cells. The former suggests a strong role for membrane and cytoplasmic viscosity in cell transit through a constricted microchannel, whereas the latter found no such role. At present, this remains an open question.

In the present study, we develop a three-dimensional computational method to simulate the transient deformation of suspended cancer cells flowing through a constricted microchannel. Our cell model takes into account the three major subcellular components: a viscoelastic membrane that represents the lipid bilayer and the underlying cell cortex, a viscous cytoplasm, and a nucleus modelled as a smaller deformable capsule. We solve the cell motion and deformation by means of an immersed boundary lattice Boltzmann method (Sui et al 2008a, b; Wang et al 2016, 2018; Lu et al 2021). Our ultimate aim is to build a minimal model which can accurately predict the transient deformation of cancer cells flowing in channels. However, validation of such a model against a wide variety of cell types is not currently possible, due to the lack of comparable experimental data on cell transit in fluid flows. As a first step towards the goal, we conduct extensive parametric studies of the effects of the rheology of the three subcellular components on cell transient deformation and compare the results with a recent experiment conducted on human leukaemia cells (Fregin et al 2019).

Our paper is organized as follows: in Sect. 2, we describe the flow problem and cell model and briefly cover the numerical method and its validation. In Sect. 3, we present simulation results from extensive parametric studies of the effects of the rheology of the subcellular components and determine the essential cell features that must be included in a minimal mechanical model to accurately reproduce the cell dynamics observed in the recent experiment. We conclude the paper and discuss the limitations and potential applications of the model in Sect. 4.

## Problem statement and computational method

### Problem description

**Fig. 1:**
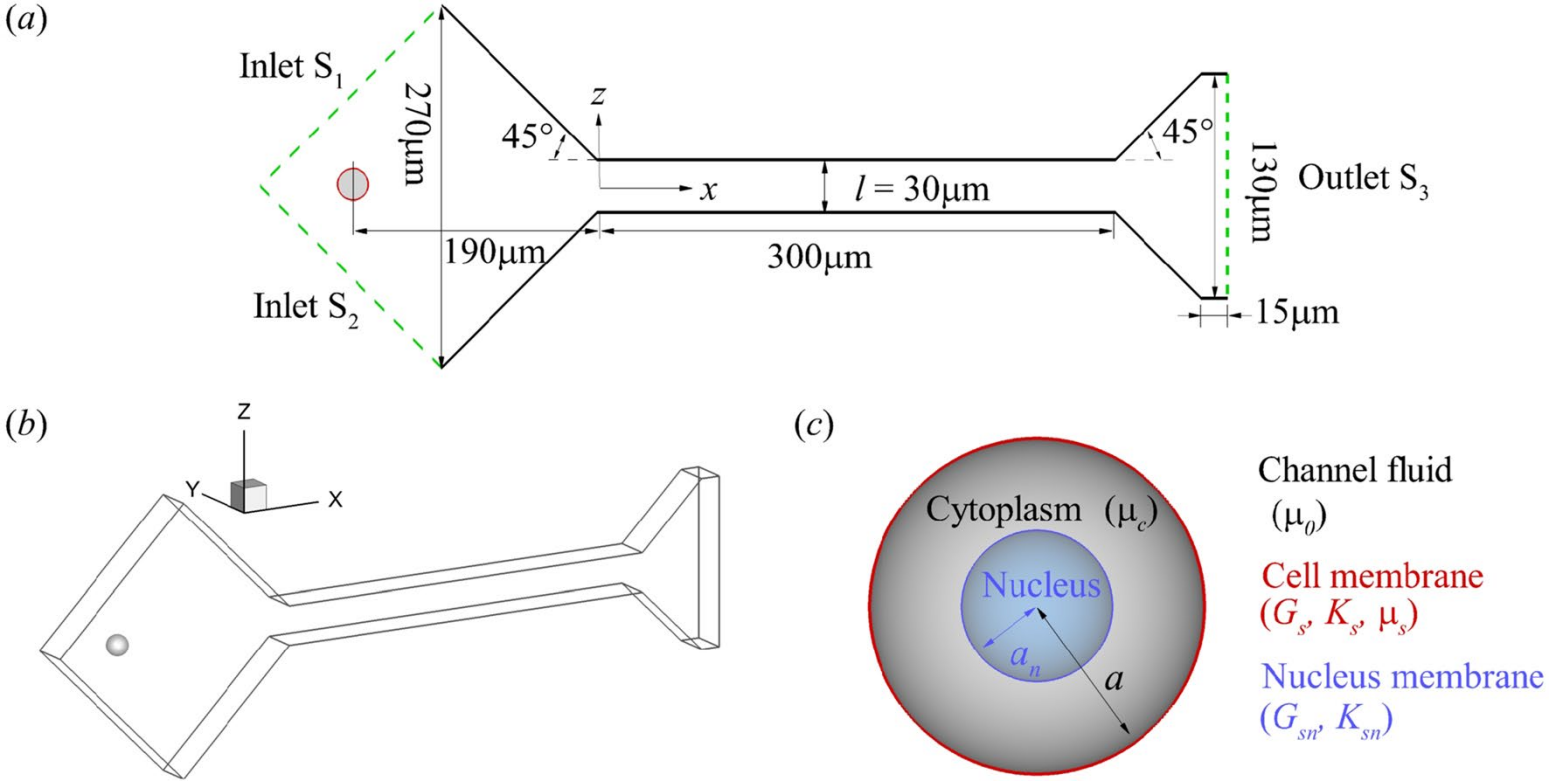
**a** Channel geometry in $x-z$ plane. **b** Geometry of the 3D computational domain. (c) Illustration of the cell mechanical model

We consider an initially spherical cancer cell flowing through a constricted channel, as shown in Fig. 1. The flow setup is taken from a recent experiment (Fregin et al 2019) conducted on human leukaemia HL-60 cells. The cell has a radius of $a=8.5\,\upmu$m and is released with zero velocity at $x=-190\,\upmu$m. It first flows through a constriction with a constant converging angle of $45^{\circ }$ and then enters a narrow straight channel with a square cross section that has a width of $l=30\,\upmu$m. The length of the straight channel is $300\,\upmu$m and it is connected to a diverging section with a constant angle of $45^{\circ }$. A three-dimensional Cartesian coordinate system is defined with the *x*-axis along the flow direction, the *y* and *z*-axes along the depth and width of the channel, and the origin at the centre of the plane which connects the converging section and the straight narrow channel (see Fig. 1a). The flow is driven by a constant pressure difference between the channel inlets and the outlet, which can be tuned in the absence of the cell to match the flow rate of the experiment. At the same flow rate, the fluid velocity along the centreline of the channel in the present simulation is almost identical to that reported by Fregin et al (2019). Due to the small volume of the cell compared with the computational domain, its effect on the flow rate is negligible. The no-slip boundary condition is imposed on all solid walls.

### Cell mechanical model

The present mechanical model for cancer cells has included the three main subcellular components: a membrane, a cytoplasm and a nucleus (see Fig. 1c). For most biological cells, the plasma membrane is supported by an underlying actin cortex which reinforces the thin lipid bilayer (Yeung and Evans 1989; Mogilner and Manhart 2018). This structure contributes to the membrane viscosity, and the elastic resistance to shear deformation and area dilatation. Here, we assume that the total stress tensor of the viscoelastic membrane is the sum of the elastic and viscous stresses:

1 $$\begin{aligned} {\varvec{\tau }}={\varvec{\tau }}^e+{\varvec{\tau }}^\nu . \end{aligned}$$

The cell membrane is assumed to be infinitely thin and its elasticity follows the strain-hardening Skalak’s (SK) law (Skalak et al 1973), with a strain energy function:

2 $$\begin{aligned} W=\frac{1}{4}G_s\left( I_1^2+2I_1-2I_2\right) +\frac{1}{4}CG_sI_2^2, \end{aligned}$$

where *W* is the strain energy density per unit undeformed surface area, $G_s$ is the surface shear elasticity modulus, $I_1$ and $I_2$ are two strain invariants with $I_1={\lambda _1^2}+{\lambda _2^2}-2$ and $I_2=(\lambda _1\lambda _2)^2-1$. Here $\lambda _1$ and $\lambda _2$ are the principal extension ratios. The membrane area dilatation modulus is $K_s=(1+2C)G_s$. For a cell membrane, the hardness parameter *C* is usually much larger than unity. The principal elastic tensions $\tau _1^e$ and $\tau _2^e$ in the membrane plane are given by

3 $$\begin{aligned} \begin{aligned} \tau _1^e=\frac{G_s\lambda _1}{\lambda _2}\left( \lambda _1^2-1+C\lambda _2^2I_2\right) ,\\ \tau _2^e=\frac{G_s\lambda _2}{\lambda _1}\left( \lambda _2^2-1+C\lambda _1^2I_2\right) . \end{aligned} \end{aligned}$$

The elastic stress tensor can be obtained from

4 $$\begin{aligned} {\varvec{\tau }}^e=\tau _1^e{\varvec{e}}_1 \otimes {\varvec{e}}_1+\tau _2^e{\varvec{e}}_2 \otimes {\varvec{e}}_2, \end{aligned}$$

where ${\varvec{e}}_1$ and ${\varvec{e}}_2$ are directions corresponding to two principal tensions.

The viscous stress of the membrane is separated into the contributions from the membrane shear viscosity $\mu _s$ and from the membrane dilatational viscosity $\mu _s^\prime$ (Barthès-Biesel and Sgaier 1985):

5 $$\begin{aligned} {\varvec{\tau }}^\nu =\mu _s[2{\varvec{D}}-tr({\varvec{D}}){\varvec{P}}]+\mu _s^\prime {\text {tr}}({\varvec{D}}){\varvec{P}}, \end{aligned}$$

where $\varvec{D}$ is the strain rate tensor of the membrane, $tr({\varvec{D}})$ is the rate of area dilatation and $\varvec{P}$ is the projection tensor of the deformed surface. In the present study, we have neglected the viscous effect due to area dilatation for simplicity. This term has been shown to be negligible for cell membranes with small area dilatation (Tran-Son-Tay et al 1984).

The bending resistance of the membrane is modelled following Helfrich’s bending energy formulation (Zhong-Can and Helfrich 1989)

6 $$\begin{aligned} E_b=\frac{k_c}{2}\int _{A_0} (2H-c_0)^2dA_0, \end{aligned}$$

where $k_c$ is the bending modulus, $A_0$ is the surface area, *H* is the mean curvature, and $c_0$ is the spontaneous curvature. A small bending resistance $k_c=0.001G_s a^2$ has been used in the present study to prevent membrane wrinkles.

The cytoplasm of the cell is modelled as a Newtonian liquid. The cell nucleus is represented by a small capsule, where a viscous fluid is enclosed by a nucleus membrane that obeys the SK law.

### Dimensionless parameters

The cell deformation is mainly determined by the following dimensionless parameters:

- The capillary number *Ca*, which compare the viscous fluid force acting on the cell to the elastic force of the cell membrane
  7 $$\begin{aligned} Ca=\frac{\mu _0 V}{G_s}, \end{aligned}$$
  where $\mu _0$ and *V* are, respectively, the average viscosity and flow speed of the fluid in the straight channel.
- The dimensionless viscosity of the cell membrane
  8 $$\begin{aligned} \eta ^*=\frac{\mu _s}{\mu _0 a}. \end{aligned}$$
- The viscosity ratio between the cell cytoplasm and channel fluid
  9 $$\begin{aligned} \lambda =\frac{\mu _c}{\mu _0}. \end{aligned}$$
- The confinement ratio between the cell diameter and channel width 2*a*/*l*.
- The size ratio between the cell nucleus and whole cell ${a_n}/{a}$, where $a_n$ is the nucleus radius.
- The flow Reynolds number *Re* evaluated in the straight channel
  10 $$\begin{aligned} Re=\frac{\rho V l}{\mu _0}, \end{aligned}$$
  where $\rho$ is the density of the channel fluid.

Since our simulation aims to reproduce quantitatively the experimental data of Fregin et al (2019), we have tried to use their experimental parameters in evaluating the dimensionless parameters in our model. First, our channel geometry and the cell size are taken directly from the experiment, thus the ratio $2a/l = 0.57$. In the experiment, the channel fluid is a phosphate-buffered saline with 1%(w/v) methylcellulose and its viscosity follows a power law

11 $$\begin{aligned} \mu =m \left( \frac{\dot{\gamma }}{\dot{\gamma _0}}\right) ^{\alpha -1}, \end{aligned}$$

where $m=0.60 {\,\text {Pa} \, s}$, $\alpha =0.64$, $\dot{\gamma }$ is the local shear rate and $\dot{\gamma _0}=1 \,\text {s}^{-1}$. The fluid density is $\rho =$1065 kg$\,\text {m}^{-3}$, which is comparable to that of the cancer cell (Zhao et al 2015). The same power law and parameters have been used in the present model. A typical flow rate is 8 nL ${\text {s}^{-1}}$, corresponding to an average flow speed in the narrow straight channel with $V=0.89$ cm ${\text {s}^{-1}}$. The average fluid viscosity $\mu _0$, calculated in the channel cross section at $x=150\,\upmu$m, is 32.5 $\text {mPa} \, \text {s}$. These lead to a flow Reynolds number of about 0.01.

At the subcellular level, the elastic moduli and viscosity of the cell membrane, cytoplasm and nucleus all remain unknown. In fact, previous studies often treated leukaemia cells as either a homogeneous solid sphere or a liquid droplet (Rosenbluth et al 2006). Therefore, the values of *Ca*, $\eta ^*$ and $\lambda$ in the present model need to be inferred by fitting simulation results to the experiment. The size of the nucleus of leukaemia HL-60 cells was also not reported in the experiment of Fregin et al (2019). However, according to Rowat et al (2013), the nucleus radius is typically 3.5-5 $\upmu$m, leading to a size ratio of $a_{n}/a$ in the range of 0.4 to 0.6.

To quantify cell deformation, we employ a deformation index (*DI*) (Fregin et al 2019) which measures the cell non-circularity

12 $$\begin{aligned} DI=1-\frac{2\sqrt{\pi A}}{P}, \end{aligned}$$

where *A* and *P* are the projected surface area and perimeter of the cell, respectively, in the symmetric $y=0$ plane.

### Numerical method and its validation

The present numerical framework is based on a well-tested immersed boundary lattice Boltzmann method (Sui et al 2008a, b; Wang et al 2016, 2018; Lu et al 2021; Lin et al 2021, 2022) and here we only provide a very brief overview. The fluid flow is solved by the three-dimensional Navier–Stokes equations using a three-dimensional nineteen-velocity (D3Q19) LBGK model. At the walls of the constricted channel, the no-slip boundary condition is applied using a second-order bounce-back scheme (Bouzidi et al 2001). A second-order non-equilibrium extrapolation method (Guo et al 2002) has been employed to impose the pressure boundary conditions at the inlets and the outlet. The interaction between the fluid and the cell is solved using the immersed boundary method of Peskin (1977). The membranes of the cell and its nucleus are discretized into flat triangular elements, following Ramanujan and Pozrikidis (1998). A finite element method (Shrivastava and Tang 1993) is used to calculate the deformation gradient tensor, the principal extension ratios $\lambda _1$ and $\lambda _2$ and the elastic stress tensor. To compute the viscoelastic stress, we follow the approach of Yazdani and Bagchi (2013), more details can be found in Appendix 1. The numerical method to calculate the bending force can be found in Appendix 1. Following Gabbanelli et al (2005), we use a truncated power-law model for the viscosity of the channel fluid surrounding the cell. To validate the power-law model, we compare the simulation result of the flow velocity between two parallel plates with the analytical solution and obtain satisfactory agreement. To account for the viscosity contrast between the cytoplasm and channel fluid, we employ a front-tracking approach (Tryggvason et al 2001; Sui et al 2010), where a colour function is used to discriminate the fluids and calculate their physical properties.

**Fig. 2:**
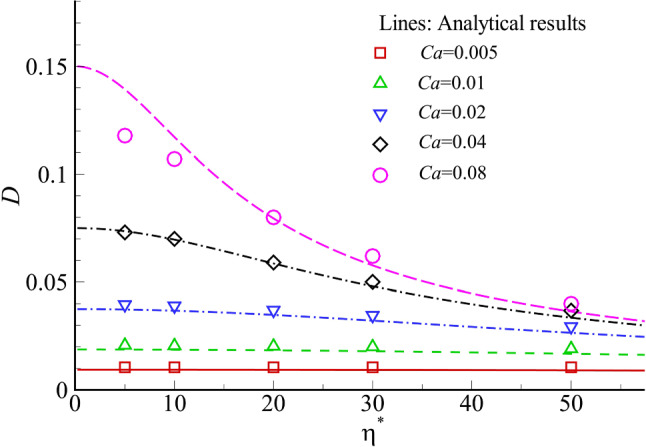
Validation of the present viscoelastic membrane model against analytical solutions. The steady Taylor shape parameter *D* of a capsule in simple shear flow at different *Ca* and $\eta ^*$ is compared between our simulations (symbols) and analytical solutions (lines) using the small deformation theory of Barthès-Biesel and Sgaier (1985). The Taylor shape parameter indicates the extent of capsule deformation and is defined as $D=(L-B)/(L+B)$, where *L* and *B* are the semimajor and semiminor lengths of the deformed capsule in the plane of shear

Our computational method for capsules with a hyperelastic membrane had been validated extensively against previous theoretical and computational results of capsules in linear shear flow (Sui et al 2008b, c) and channel flows (Wang et al 2016, 2018). Here, we validate the model for membrane viscosity by considering the deformation of a spherical capsule with a viscoelastic membrane in a Newtonian shear flow. The flow is in the Stokes regime and the capsule membrane mechanics follows Eqs. (2) and (5). Two main dimensionless parameters that determine capsule deformation are: the dimensionless membrane viscosity $\eta ^*=\mu _s/\mu _0 a$ and the capillary number $Ca=\mu _0 \dot{\gamma } a/G_{s}$, where $\dot{\gamma }$ is the shear rate. In Fig. 2, we compare our simulation results with predictions of the small deformation theory of Barthès-Biesel and Sgaier (1985). Good agreement has been obtained when the Taylor deformation parameter of the capsule is less than 0.1. Membrane viscosity has restricted the overall deformation of the tank-treading capsule in the linear shear flow.

For the present problem of a cancer cell flowing through a constricted channel, we have conducted mesh convergence study for both the fluid and cell membrane grids. The fluid grid size that is finally chosen is $\Delta x=0.0156l$. The membranes of the cell and its nucleus have been discretized into 8192 flat triangular elements connecting 4098 nodes, with a maximum element edge length of $\Delta L_c\sim 0.0215l$. Using a finer fluid grid of $\Delta x=0.0125\,l$ or increasing the number of membrane elements to 32768 does not lead to any visible change in the cell’s flow trajectory or deformed shapes (not shown). We have also examined the effect of the initial axial position of cell and found that the cell transient deformation in the region of interest, i.e. $x \ge -60\,\upmu$m, is almost identical when the cell is released from $x \le -160\,\upmu$m.

## Results and discussion

In this section, we conduct extensive parametric studies of the effects of mechanical properties of the cell membrane, cytoplasm and nucleus on the transient cell deformation in the constricted microchannel. We consider cell models with increasing complexity and compare the simulation results, in terms of the spatial evolution of the cell deformation index and cell profiles, with the recent experiment of Fregin et al (2019). The comparison enables us to identify a minimal cell mechanical model and estimate values of associated parameters that can accurately reproduce the transient cell deformation in the constricted channel.

### A cell with a hyperelastic membrane

#### Estimation of cell membrane elasticity

We start from a simple cell mechanical model where the membranes of the cell and its nucleus are both purely hyperelastic that follow the SK law. We do so mainly for three reasons. Firstly, the model is the simplest; it can be solved through well-established numerical approaches such as the boundary element method or immersed boundary method. Secondly, in the experiments of Fregin et al (2019), the leukaemia HL-60 cell has reached an apparent steady profile at the end of the narrow straight channel. The cell cytoplasm is therefore largely in solid translation and viscous effects from cell subcellular components will not play significant roles in determining the cell steady shape. A hyperelastic cell model that accurately accounts for the cell elasticity should be sufficient to predict the cell steady shape and enable estimations of the cell membrane elastic moduli $G_s$ and $K_s$. Note that the values of the two parameters of leukaemia HL-60 cells have not been reported in any previous study. Finally, the hyperelastic model enables us to identify the unique effect of membrane elasticity on cell deformation.

We cover a wide range of $G_s$ and $K_s$ that correspond to $0.1\le Ca \le 5$ and $1 \le C \le 50$. In Sect. 3.1, unless otherwise specified, we assume that the viscosity of the cell cytoplasm is identical to that of the channel fluid ($\lambda =1$), and the nucleus has a size ratio of $a_n/a=0.5$. The elastic moduli of the cell nucleus membrane are also assumed to be twice those of the cell membrane, to represent the fact that a cell nucleus is generally stiffer than the whole cell. Effects of those parameters on cell deformation will be studied in later sections.

**Fig. 3:**
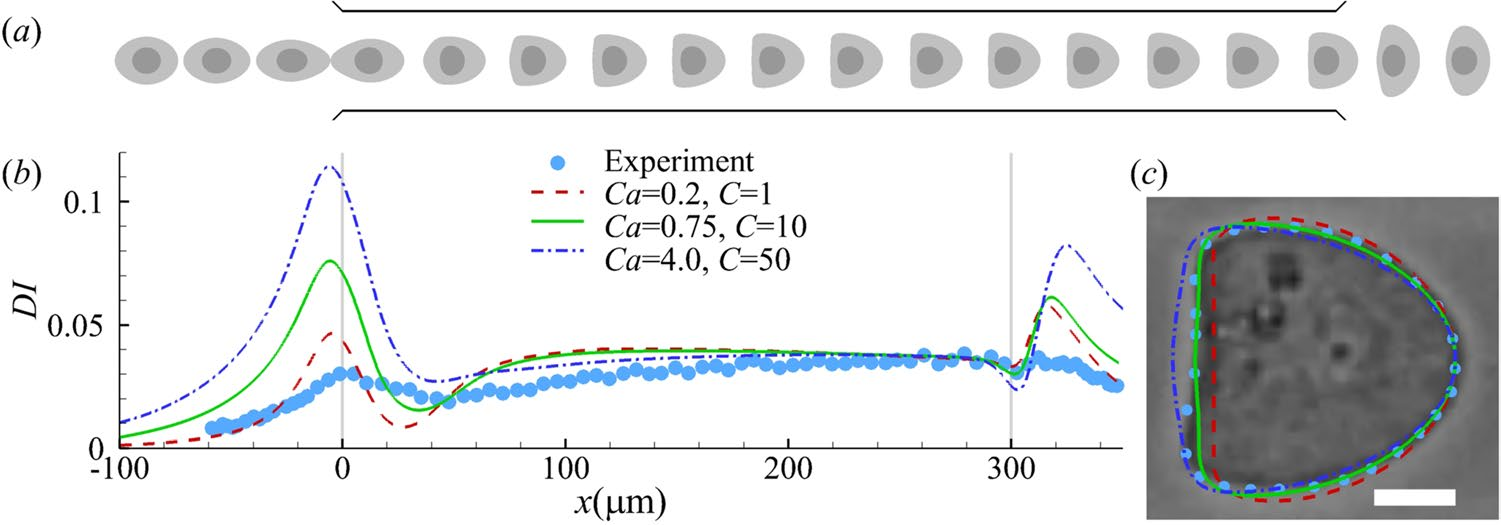
**a** Instantaneous profiles of a modelled cell with hyperelastic membranes (SK law with $C=10$) flowing through a constricted channel at $Ca=0.75$. **b** Cell deformation index as a function of the axial position of the cell mass centre. All three combinations of *Ca* and *C* can lead to good agreement in the steady *DI* of the cell in the narrow straight channel between numerical simulation (lines) and the experiment of Fregin et al (2019) (symbols). In the experiment, the cell reaches steady deformation at around $x=250\,\upmu$m. In simulations with the hyperelastic membrane model, steady deformation is reached earlier, before $x=200\,\upmu$m. **c** Comparison of the cell steady profiles of the three numerical simulations in (**b**) with the experiment when the cell mass centres are at $x=274\,\upmu$m. In **c** the scale bar represents 5 $\upmu$m

An example of the cell transient profiles in the constricted channel is shown in Fig. 3a, for a modelled cell with SK membranes with $C=10$ at $Ca=0.75$. The cell is firstly elongated in the flow direction in the converging part of the channel, and the elongation is at the maximum when the cell is entering the narrow straight channel. Inside the narrow channel, the cell gradually develops into a steady bullet shape at about $x=150\,\upmu$m, under the effect of fluid shear. In the diverging part of the channel, the cell is compressed along the flow direction. The cell deformation history can be readily understood by features of the undisturbed flow inside the channel.

Figure 3b depicts the spatial evolution of the deformation index for three sets of *Ca* and *C* values. Interestingly, all three combinations of parameters capture the steady deformation index accurately. However, when comparing the steady cell cross-sectional profiles (in the symmetric plane at $y=0$), we find that the parameter combination of $Ca=0.75$ and $C=10$ gives the best agreement with the experimental profile (Fig. 3c). At the best fit, the cell membrane area has increased by 4%. The present study therefore suggests that compared with the deformation index, the cell shape serves as a better indicator when comparing a simulation result with the experiment. In the present setup, the parameters of $Ca=0.75$ and $C=10$ correspond to cell membrane elastic moduli values of $G_s=0.39 \,{\text {mN}\,\text {m}^{-1}}$ and $K_s=8.10 \,{\text {mN}\,\text {m}^{-1}}$. Neither has been reported in any previous study of leukaemia HL-60 cells. The inferred membrane shear elasticity is close to that of neutrophils which has $G_s=0.35 \,{\text {mN}\,\text {m}^{-1}}$ (Dong and Skalak 1992).

#### Effect of membrane elasticity on cell deformation

**Fig. 4:**
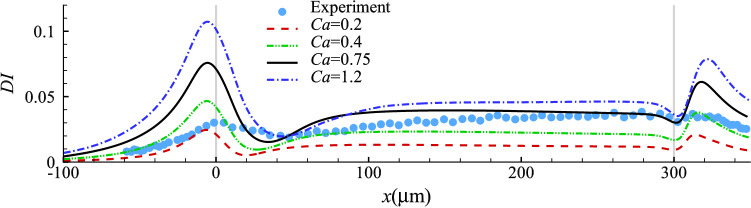
Cell deformation index as a function of the axial position of the cell mass centre at different *Ca* with $C=10$. The experimental result is from Fregin et al (2019) and is shown in symbols

To vary the shear and dilatation moduli of the cell membrane simultaneously, we vary the capillary number *Ca* while keeping the membrane hardness parameter *C* constant. The nucleus-to-cell membrane shear elastic moduli ratio is also kept as $G_{sn}/G_s=2$. Figure 4 presents the evolution of cell deformation index along its axial position for $Ca=0.2 \sim 1.2$ with $C=10$. It is seen that the maximum deformation of the cell occurs a very short distance before the narrow channel at about $x=-6$ $\upmu$m, irrespective of the cell membrane elasticity. The maximum deformation decreases with the membrane elasticity. After entering the narrow channel, the cell deformation index drops sharply due to a rapid decrease in flow extension. It then evolves to a steady value that is determined by the shear stress of the channel fluid and the cell membrane elasticity.

As shown from Fig. 3b, although the steady cell deformation index can be reproduced by several combinations of *Ca* and *C*, we find it impossible for any combination to capture the transient cell deformation further upstream. The maximum cell deformation in the experiment is much lower than that of the simulation, and it takes place further downstream, approximately at the entrance of the straight channel. Furthermore, compared with the experiment, the time required for the simulated cell deformation index to drop from the peak to the following trough is also much shorter (not shown). These features suggest that the present mechanical model has under-represented viscous dissipation within the cell during its transient deformation. The results are not surprising. Several sources can contribute to the viscous dissipation of the cell, such as the cell membrane and cytoplasm. We will first consider the effect of the cytoplasm viscosity in Sect. 3.1.3.

#### Effect of cytoplasm viscosity

**Fig. 5:**
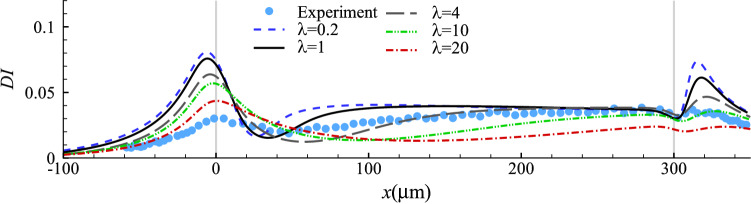
Cell deformation index as a function of the axial position of its mass centre with different values of viscosity ratio $\lambda$ at $Ca=0.75$ and $C=10$. Symbols are experimental results (Fregin et al 2019)

In our numerical tests of this section, we keep the parameter values that have led to the best fit with the experiment in the steady cell profile while adjusting the cell cytoplasm viscosity. The spatial evolution of the deformation index of the cells with a wide range of cytoplasm viscosity ($0.2 \le \lambda \le 20$) is presented in Fig. 5, where several interesting observations can be made. Firstly, higher cytoplasmic viscosity reduces the maximum deformation of the cell in the constriction. Secondly, it slows down cell deformation, delaying the onset of the maximum cell deformation in the constriction and the approach to steady deformation in the narrow straight channel. Note that both features are needed to improve the agreement between the simulation and experiment (see Fig. 3b). However, by varying $\lambda$ alone it is impossible to achieve a good agreement between the model prediction and experiment in the cell deformation along the entire length of the microchannel.

It has been suggested in previous studies that the cytoplasm of biological cells, e.g. leukocytes, may be shear-thinning, because it is a suspension of filaments and organelles (Tsai et al 1993; Marella and Udaykumar 2004). Here one may wonder if a shear-thinning cytoplasm model would improve the agreement between the model prediction and experiment. From simulation results (not shown), we find that the volume-average strain rate of the cytoplasm peaks when the cell is near the entrance of the narrow straight channel, due to the rapid elongation of the cell, and then decreases as the cell flows downstream. If the cytoplasm was shear-thinning, its viscosity would be at a minimum near $x=0$ and then increase down the straight channel. However, Fig. 5 suggests that to match the cell deformation along the entire length of the microchannel in the experiment, a higher cytoplasmic viscosity with $\lambda > 20$ is needed at $x=0$, and the cytoplasm viscosity should decrease quickly when the cell flows downstream so that it can reach the steady shape at around $x=250\,\upmu$m. Therefore, cytoplasmic shear-thinning would not remedy the inadequacies of the model shown in Fig. 5.

### A cell with a viscoelastic membrane

The poor performance of the hyperelastic cell model in predicting cell transient deformation presented in Sect. 3.1 is not surprising. The membrane of biological cells generally consists of a viscous lipid bilayer with cholesterols and various proteins. For RBCs, previous studies (Hochmuth et al 1979; Tran-Son-Tay et al 1984; Tomaiuolo and Guido 2011; Prado et al 2015) have shown the important role of membrane viscosity in determining the relaxation time of the whole cell. Relatively few studies have been done on the membrane viscosity of other cells, but modelling of neutrophils has suggested that membrane viscosity is essential to explain the time-dependence of cell entry into a micropipette under aspiration (Drury and Dembo 2001; Herant et al 2003).

In this section, we consider a cell with a viscoelastic membrane. The membrane elasticity follows the same SK law that is used in Sect. 3.1, and we add the membrane viscosity using Eq. (1). In our simulations in this section, we adjust the cell membrane viscosity, while keeping the values of all other parameters that have led to the best fit with the experiment in the steady cell profile in Sect. 3.1. Note that the cell membrane viscosity does not affect the steady cell shape.

#### Cell membrane with a constant viscosity

**Fig. 6:**
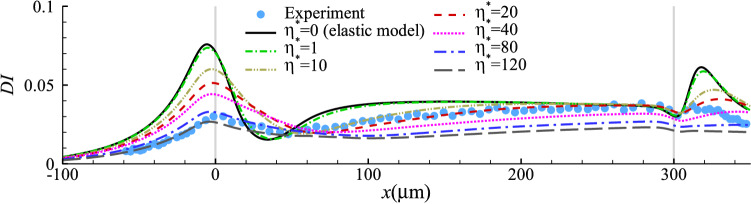
Cell deformation index as a function of the axial position of the cell mass centre for different cell membrane viscosity $\eta ^*$ at $Ca=0.75$ and $C=10$

We first employ a Newtonian membrane viscosity model and consider a cell with different levels of constant membrane viscosity. Figure 6 presents the spatial evolution of the deformation index of cells with $1 \le \eta ^*\le 120$. The results indicate that the effect of cell membrane viscosity on cell transient deformation is generally similar to that of the cell cytoplasm viscosity. When comparing the simulation results with the experiment, we can find that near the entrance of the narrow straight channel, a good agreement with the experiment requires a high viscosity: $80 \le \eta ^*\le 120$, while approaching the channel exit, a much smaller value of $\eta ^*\le 20$ is needed for the cell to reach its steady shape. The inconsistency suggests that a Newtonian viscosity model is not sufficient to account for the complexity of the cell membrane rheology in the experiment.

#### Cell membrane with shear-thinning or -thickening rheology

We then consider a cell with its membrane following shear-thinning or -thickening rheology. The empirical model of Drury and Dembo (2001) has been used for the membrane viscosity:

13 $$\begin{aligned} \mu _s=\mu _1\left( 1+\frac{{\bar{\gamma }}_m}{\gamma _0}\right) ^n. \end{aligned}$$

**Fig. 7:**
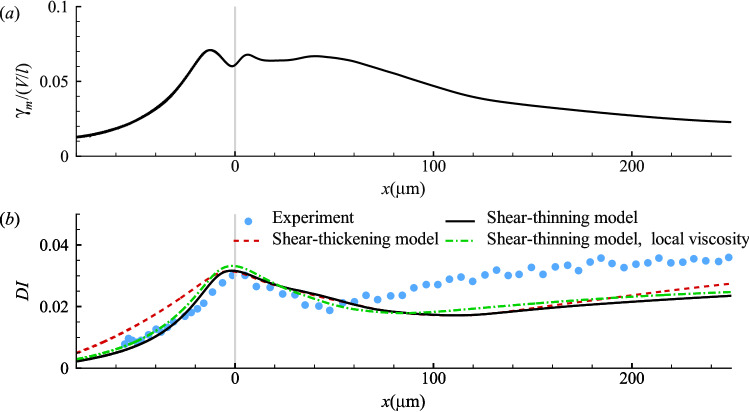
**a** The membrane area-average strain rate ${\bar{\gamma }}_m$ as a function of the cell position at $Ca=0.75$, $C=10$, $\eta ^*=40$. **b** Comparison of the deformation index of cells with a shear-thinning or -thickening membrane. The parameters in the shear-thinning model are $\mu _1 / \mu _0 a=400$, $\gamma _0 / (V/l)=0.0075$, $n=-0.8$, and in the shear-thickening model $\mu _1 / \mu _0 a=19$, $\gamma _0 / (V/l)=0.005$ and $n=0.8$. The dash dot line is the prediction of the shear-thinning model with $\mu _s$ determined from the local membrane strain rate. The membrane elasticity follows the SK law with $Ca=0.75$, $C=10$. The experimental result of Fregin et al (2019) is shown in symbols

The constants $\mu _1$ and $\gamma _0$ represent a zero-shear viscosity and a characteristic strain rate, respectively. The sign of the power exponent *n* determines shear-thinning or -thickening. The strain rate ${\bar{\gamma }}_m=\sqrt{2tr({\varvec{D}}^2)}$ is averaged over the entire cell membrane. One example of ${\bar{\gamma }}_m$ is shown in Fig. 7a for a cell with a constant membrane viscosity of $\eta ^*=40$. We find that ${\bar{\gamma }}_m$ peaks near the entrance of the narrow straight channel, due to the rapid cell elongation and its subsequent adaption to shear deformation. The membrane strain rate then decreases when the cell is flowing down the narrow channel and approaching its steady bullet shape. In Eq. (13), we have assumed that the membrane viscosity is a global property that depends on the area-averaged membrane strain rate. In principle $\mu _s$ can be determined locally, but the former approach is more computationally stable, enabling us to explore a much wider parametric space. A comparison between the predictions of the two methods is presented in Fig. 7b for a shear-thinning membrane, and the results seem to be very similar.

Using Eq. (13) over a wide range of parameters, we find it impossible to bring the model predictions into close agreement with the experiment. With a shear-thinning model, since the membrane strain rate generally drops when the cell flows down the narrow straight channel, the cell membrane viscosity increases. Figure 6 shows, on the other hand, that the membrane viscosity would need to decrease after the cell enters the narrow channel in order to capture the higher *DI* in experiments. One example of our simulations is shown in Fig. 7b using a solid line, for a cell with $\mu _1 / \mu _0 a=400$, $\gamma _0 / (V/l)=0.0075$ and $n=-0.8$. The shear-thinning membrane approximately reproduces the maximum deformation observed in the experiment; however, it underpredicts *DI* in the straight channel downstream due to the high membrane viscosity.

In view of the above, a shear-thickening membrane model seems to be more promising in that the cell membrane viscosity will generally drop when the cell is flowing downstream the straight channel. However, the membrane strain rate remains high in the straight channel for a long distance, till $x=60 \,\upmu$m as shown in Fig. 7a. Therefore, the decrease of the membrane viscosity is not quick enough for a good agreement with the experiment. The dash line in Fig. 7b is for a cell with shear-thickening membrane with $\mu _1 / \mu _0 a=19$, $\gamma _0 / (V/l)=0.005$ and $n=0.8$. With the maximum deformation approximately matching the experiment, the model cell is unable to respond to the flow as quickly as needed after it enters the straight channel. In addition, the shear-thickening model also overpredicts *DI* in the upstream converging channel.

#### A phenomenological model for membrane viscosity

Based on the observation from Fig. 6 that the membrane viscosity should behave differently when the cell is in the converging and straight sections of the channel, and the fact that the cell is subject to distinct elongational and shear effects in the two channel sections, we propose a simple phenomenological model to describe the cell membrane viscosity. In the model, the membrane viscosity depends on whether it is being stretched or sheared:

14 $$\begin{aligned} \mu _s=(1-\epsilon )\mu _s^s+\epsilon \mu _s^e. \end{aligned}$$

The constants $\mu _s^s$ and $\mu _s^e$ represent the membrane shear and extensional viscosity, respectively. The term $\epsilon$ represents the flow type parameter of the background flow in the symmetric plane $y=0$: $\epsilon =(\mid \dot{\gamma }\mid -\mid \omega \mid )/(\mid \dot{\gamma }\mid +\mid \omega \mid )$, where $\mid \dot{\gamma }\mid$ and $\mid \omega \mid$ are the magnitudes of the fluid strain rate tensor $\dot{\gamma }=\nabla {\varvec{u}}+\nabla {\varvec{u}}^{\text {T}}$ and the fluid vorticity tensor $\omega =\nabla {\varvec{u}}-\nabla {\varvec{u}}^{\text {T}}$, respectively (Fuller and Leal 1980; Patil et al 2006). The flow type parameter is an average along $-a \le z \le a$ at the $x-$axis position corresponding to the mass centre of the cell. For simple shear or extensional flow, $\epsilon =0$ or 1, respectively.

Basing the membrane viscosity on the flow-type parameter follows from an extensive rheological literature on the response of polymeric liquids to different flow types (Fuller and Leal 1980; Singh and Leal 1994; Patil et al 2006). Despite the heterogeneous structure of the cell membrane, we may liken its viscosity to that of a polymeric liquid, which exhibits distinct extensional and shear rheologies. The two different modes of deformation elicit different conformational changes in the polymer chains, with extensional flow typically causing chain stretching and alignment and provoking a much stronger mechanical response than shear flow. The ratio of the extensional and shear viscosity is the Trouton ratio *Tr*, which is always three for a Newtonian fluid. However, *Tr* can be orders of magnitude higher for polymeric fluids in an effect commonly called “strain hardening” (Bird et al 1987).

**Fig. 8:**
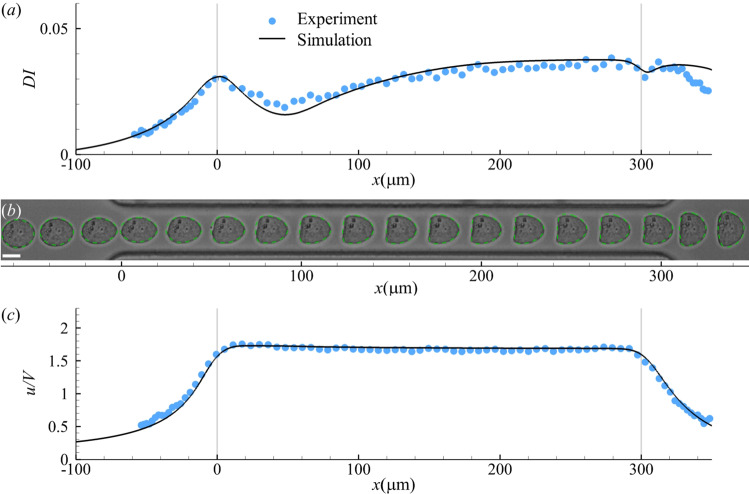
Comparisons of **a** cell deformation index, **b** instantaneous profiles, and **c** cell flow speed obtained from the present simulation and experiment (Fregin et al 2019). In **b** the scale bar represents 10 $\upmu$m. In simulation, the cell membrane viscosity follows Eq. (14) with $\mu _s^{e}/\mu _0 a=100$, $\mu _s^{s}/\mu _0 a=20$. The membrane elasticity follows the SK law with $Ca=0.75$, $C=10$

We find excellent agreement with the experiment when using Eq. (14) with $\mu _s^{e}/\mu _0 a=100$ and $\mu _s^{s}/\mu _0 a=20$. This is demonstrated in Fig. 8 using the cell deformation index, transient profiles, and cell velocity. In the experimental images, the cell appears slightly off-centre before entering the straight narrow channel (Fig. 8b). We have tested the effect of a small initial offset in our simulations, up to 2*a* along the $-z$ direction, and found the cell behaviour little affected. All the results of Fig. 8 and below use an initial offset of *a*.

Note that the membrane viscosity of leukaemia cells has not been reported previously. In Fig. 8, the dimensional cell membrane viscosity $\mu _s^{s}$ and $\mu _s^{e}$ are 5.5 and $27.5\,{\upmu \text {N} \, \text {s}\,\text {m}^{-1}}$, respectively. These are comparable to the membrane viscosity of $8.5\,{\upmu \text {N} \, \text {s}\,\text {m}^{-1}}$ reported for granulocytes (Evans and Yeung 1989), but are much higher than that of a RBC membrane (Evans and Hochmuth 1976; Hochmuth et al 1979; Tran-Son-Tay et al 1984). Interestingly, we notice that previous experiments had reported distinct values of membrane viscosity when RBCs were subjected to shear or extensional flow. In simple shear flow (Tran-Son-Tay et al 1984), the RBC membrane viscosity was found to be on the order of $0.1\,{\upmu \text {N} \, \text {s}\,\text {m}^{-1}}$. Significantly higher values, on the order of $1\,{\upmu \text {N} \, \text {s}\,\text {m}^{-1}}$, were reported when cells were stretched during micropipette aspiration (Evans and Hochmuth 1976; Hochmuth et al 1979). Therefore, our results suggest that the leukaemia cell resembles the RBC in how its membrane viscosity depends on the mode of deformation. Of course, the underlying mechanisms may differ. For example, the spectrin network of the RBC membrane may behave differently from the leukaemia cell membrane, especially under the large strains typical of micropipette aspiration.

**Fig. 9:**
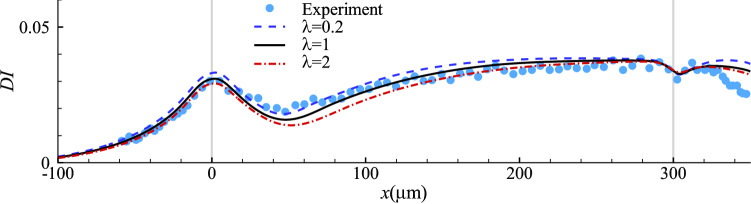
Cell deformation index as a function of cell axial location with different viscosity ratios. Other parameters are the same to those of Fig. 8

Since the cytoplasmic viscosity of leukaemia HL-60 cells is not known, in Fig. 8, we have used the baseline value $\lambda =1$. Figure 9 explores the effect of $\lambda$. In the range of $0.2\le \lambda \le 2$, the dependency of cell deformation on the cytoplasm viscosity is not strong. A unity viscosity ratio seems to give the best fit. In the present setup, this corresponds to a cytoplasm viscosity of 32.5 ${\text {mPa}\,\text {s}}$. It has been found in previous studies that the effective cytoplasmic viscosity of biological cells ranges roughly from 10 to 100 ${\text {mPa}\,\text {s}}$ (Luby-Phelps 1999; Mogilner and Manhart 2018), which is consistent with the value inferred from the present study for the leukaemia HL-60 cell.

### Effect of the cell nucleus

So far in our simulations we have fixed the nucleus size at $a_{n}/a=0.5$. We further assume that the nucleus membrane is hyperelastic, following the SK law, but stiffer than the cell membrane with $G_{sn}=2G_{s}, C=10$. Here, we test the effects of the nucleus size and its membrane stiffness on the transient cell deformation in the constricted channel. For the cell membrane and cytoplasm, we use the same models and associated parameters of Fig. 8.

**Fig. 10:**
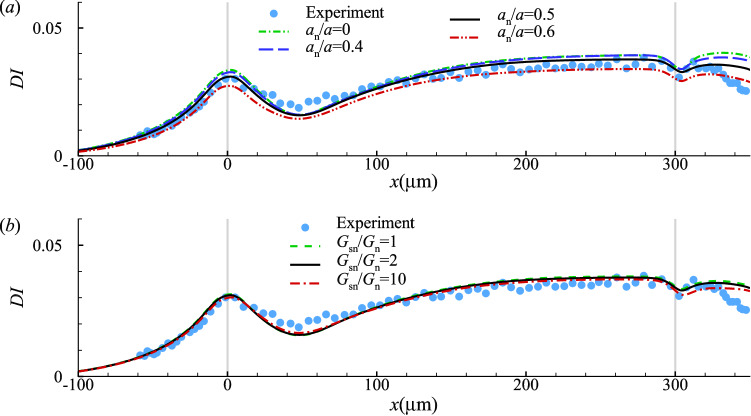
Effect of **a** cell nucleus size, and **b** nucleus membrane shear elasticity on evolution of the cell deformation index. Other parameters are the same to those of Fig. 8

Not surprisingly, a larger cell nucleus decreases the overall deformation of the cell (Fig. 10a). However, in the practical range of $0.4 \le a_{n}/a \le 0.6$ for the leukaemia HL-60 cell considered, the effect is small. The effect of membrane elasticity of the cell nucleus for a cell with $a_{n}/a=0.5$ is shown in Fig. 10b. A nucleus membrane that is one order of magnitude stiffer than the cell membrane does not significantly reduce the cell deformation in the present case, because the nucleus of the HL-60 cell is not very large and therefore does not sustain much deformation during the cell’s transit. Indeed, the present results suggest that when $a_{n}/a \le$0.5, it is not essential to account for the cell nucleus in the mechanical model.

Nevertheless, it has been known that cancer cells typically have a larger nucleus than healthy cells. We expect that the effect of the nucleus membrane stiffness on the overall cell deformation in a microfluidic channel will become increasingly significant with the size of the nucleus. A large and stiff cell nucleus could be the dominant factor in limiting the cell deformation, in particular in small channels with the cross-sectional dimension being similar to that of the cell.

## Conclusions and limitations

The present study aims to determine the minimum set of attributes that must be included in a mechanical model to quantitatively predict the transient deformation of suspended cancer cells flowing through a constricted channel. To achieve this goal, we have conducted extensive and systematic numerical simulations, using a range of models with increasing complexity, and compared the simulation results with a recent experiment where the transient profiles of a human leukaemia HL-60 cell in a constricted microchannel were clearly recorded. We find that the hyperelastic cell membrane model, using the SK law, can only recover the steady-state deformation of the cell in the straight channel. Excellent agreement with the experiment in transient cell dynamics can be achieved by properly accounting for the membrane viscoelasticity. Specifically, the membrane viscosity of the cell in elongational deformation should be higher than that when the cell deformation is shear-dominant, by a factor of about five. The cell nucleus, with a stiffer membrane than the cell membrane, tends to reduce the overall cell deformation. However, its effect is small when the nucleus is not too large, i.e. $a_{n}/a\le 0.6$ in the present setup.

A limitation of the present study is that the model has only been validated against a leukaemia cell line. As a general model, of course, it needs to be tested on other cell types and in other flow geometries. Such broad validation is not possible at present, due to the lack of quantitative experimental data on the transit of suspended cells in a fluid medium. Other experiments such as micropipette aspiration of cells, cell squeezing through narrow constrictions, or cell migration on substrates, in ECM and through confinement, involve cell response that might fundamentally differ from the present situation where the cell is carried passively by the flow without touching the solid wall. To use such data for validation, the model must be equipped with additional features, to consider, for instance, the stress fibres in adherent, active cell cortex deformation, cell-ECM and cell-wall interactions (Borau et al 2011; Peng et al 2013; Tozluoğlu et al 2013; Allena et al 2015; Zhu and Mogilner 2016; Lee et al 2017; Lykov et al 2017; Maxian et al 2020; Balogh et al 2021; Campbell and Bagchi 2021), which are beyond the scope of the present study. Our model has considered the cytoplasm as a viscous liquid and is therefore mainly suitable for suspended cells, where the cytoskeleton consists mostly in the cell cortex underlying the plasma membrane, which is represented by our membrane model. For cells with a strong cytoskeleton linking the cell membrane and nucleus, such as the many types of solid tumour cells, it may be more suitable to model the cytoplasm as a viscoelastic solid.

Regarding practical applications, the present computational model has the potential of inferring the mechanical properties of subcellular components of suspended cancer cells from their transient flow-induced deformation. By fitting the steady deformed cell profile to computational results, one can obtain the cell membrane shear and dilatational moduli. The spatial evolution of the cell deformation will enable one to estimate the viscosities of the cell membrane and cytoplasm. These have been demonstrated in the present study on the HL-60 cells. For further validation, however, our method of inverse analysis should be tested against cell types for which the parameters to be inferred are already known. At present, no such benchmark experimental data are available in the literature. Thus validated, the computational model may facilitate the design and optimisation of microfluidic devices for mechanical characterization or sorting of suspended cancer cells.

## Figures and Tables

**Fig. 11:**
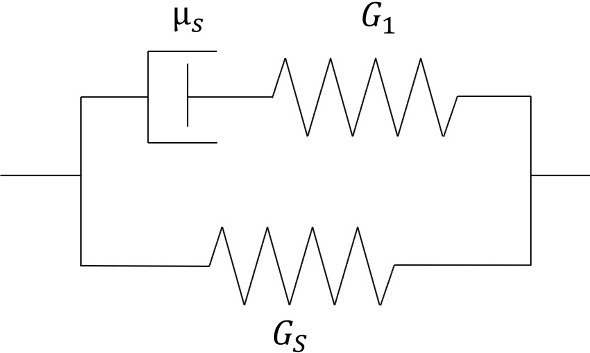
Schematic diagram of the viscoelastic membrane model

## Appendix 1: Numerical method for viscoelastic stress

The model for the membrane viscoelastic stress, Eq. (1), assumes that the elastic and the viscous terms are in parallel, and the total stress is the sum of the elastic and the viscous contributions. Previous studies had found that directly solving Eq. (1) leads to numerical instabilities due to the time derivatives of strains (Yazdani and Bagchi 2013; Li and Zhang 2019). To resolve this problem, we have adopted the methodology of Yazdani and Bagchi (2013) and employed a modified mechanical system to approximate Eq. (1). The method is briefly described here, and details can be found in pertinent literature (Yazdani and Bagchi 2013; ABAQUS/Standard Theory Manual 2002).

The modified mechanical system is illustrated in Fig. 11. Compared with the system of Eq. (1), an additional elastic term, with the stiffness $G_1$, is added in series with the viscous term to form a Maxwell element. The total viscoelastic stress is related to the strain history by a time-dependent shear relaxation modulus *G*(*t*) that is expressed with a Prony series:

15 $$\begin{aligned} G(t)=G_s+G_1e^{-t/\alpha _1}, \end{aligned}$$

where $\alpha _1=\mu _s/G_1$ is the relaxation time of the Maxwell element. When $G_1$ is sufficiently large, *G*(*t*) approaches $G_s$, and the modified mechanical system of Fig. 11 can represent Eq. (1) (Yazdani and Bagchi 2013).

The total viscoelastic stress tensor ${\varvec{\tau }} (t)$ can be written as

16 $$\begin{aligned} \begin{aligned} {\varvec{\tau }} (t)&={\varvec{\tau }}^{dev}_0 (t) + SYM\left[ \int _0 ^t \frac{{\dot{G}}(s)}{G_0} {\varvec{F}}_t^{-1}(t-s) \right. \\&\quad\left. \cdot {\varvec{\tau }}^{dev}_0 (t-s) \cdot {\varvec{F}}_t(t-s) ds\right] +{\varvec{\tau }}^{vol}(t). \end{aligned} \end{aligned}$$

The term ${\varvec{\tau }}^{dev}_0(t)$ represents the instantaneous deviatoric stress, which is equivalent to the deviatoric part of ${\varvec{\tau }}^e$ in Eq. (4), with $G_s$ replaced by the instantaneous shear modulus that can be calculated as $G_0=G(t=0)=G_s+G_1$. The term $\textbf{F}_t(t-s)$ is the deformation gradient between times $t-s$ and *t*. The operator *SYM* enforces the symmetry of the stress tensor. Since we have neglected the viscous effect due to membrane area dilatation, the membrane volumetric stress ${\varvec{\tau }}^{vol}(t)$ is equivalent to the volumetric part of the elastic stress tensor in Eq. (4). Equation (16) is integrated forward in time, following the approach detailed in ABAQUS/Standard Theory Manual (2002).

## Appendix 2: Numerical method for the bending force

The bending force density derived from the bending energy formulation Eq. (6) is (Zhong-Can and Helfrich 1989; Guckenberger and Gekle 2017)

17 $$\begin{aligned} \begin{aligned} {\varvec{f_b}}=k_c[(2H+c_0)(2H^2-2\kappa _g-c_0 H)\\ +\nabla _{s}\cdot \nabla _{s}(2H-c_0)]{{\varvec{n}}}, \end{aligned} \end{aligned}$$

where $\kappa _g$ is the Gaussian curvature, and ${\varvec{n}}$ is the outwards unit normal vector. The curvatures and the normal direction at each node of the cell membrane are calculated using a quadratic surface fitting (Garimella and Swartz 2003; Yazdani and Bagchi 2012)

18 $$\begin{aligned} z'=ax'^2+bx'y'+cy'^2+dx'+ey', \end{aligned}$$

where $(x', y', z')$ represents a local coordinate system with the origin being the membrane node of interest, and the $z'-$axis aligning with the estimated normal direction. To fit the coefficients in Eq. (18), one-ring neighbouring points have been used with a least-square method. Then, the curvatures, *H* and $\kappa _g$, and the normal unit vector ${\varvec{n}}$ can be calculated from

19 $$\begin{aligned}{} & {} \begin{aligned}&H =\frac{a+c+ae^2+cd^2-bde}{(1+d^2+e^2)^{3/2}},\\&\kappa _g=\frac{4ac-b^2}{(1+d^2+e^2)^2}, \end{aligned} \end{aligned}$$

20 $$\begin{aligned}{} & {} {{\varvec{n}}}=\frac{1}{(d^2+e^2+1)^{1/2}}[-d, -e, 1]^T. \end{aligned}$$

To discretize the Laplace–Beltrami operator $\nabla _{s}\cdot \nabla _{s}$, we follow the approach of Meyer et al (2003).
